# Monthly Summary

**Published:** 1877-11

**Authors:** 


### MONTHLY SUMMARY.

---

*Advertising Dentists.*—That all the advertising dentists are not in America, is shown by the card of "P. A. Stevens, England, Chemist and Dentist," who is "sole proprietor and maker of a silvery white gutta percha enamel, for stopping decayed teeth." He is prepared to supply wholesale houses.

*Decline of Mechanical Dentistry.*—Prof. Barker, in a paper read before the Pennsylvania State Dental Society, takes up the subject of Mechanical Dentistry and considers its "decline inevitable and appropriate." He thinks it is neither pride, at being considered above a mechanician, nor conceit, that is at the bottom of this decline, but a deeper feeling than either of these. "Every dental substitute," says he, "introduced by the intelligent and thoughtful operator, is a source of mortification that the art and science he is practicing has failed, either through the patient's neglect, or the dentist's want of skill, to preserve organs that were designed to last the life-time of the individual." He further thinks the idea of restoring facial expression founded in error, at least so far as complete replacement of the position of the muscles is concerned, and thinks that the cause of this failure, which is notable enough in many cases, lies deeper than the simple removal of the teeth, and consequent absorbtion of the alveoli. He attributes the change due to "modified functional activity," and to organic structural change also, produced by the mutilation of those parts by the removal of the tooth and the absorbtion of their bony sockets. The first of thesecauses—*i e.*—functional activity, he thinks is interfered with, first, by the shock of extraction; second, the increased functional activity of absorbents necessary to carry off the processes, now foreign bodies; third, the more or less entire obstruction and obliteration of the arteries in the parts, and a destruction of the capillary vessels. All these causes acting on the vaso-motor nervous system of these structures he thinks will account for the atrophied condition, imperfect muscular contraction, and want of co-ordination on the part of these muscles." Dentists should "turn to mechanical dentistry with sorrow and shame, as the medical man does to the barber for the wig for his hairless patient, or to the instrument maker for the tin horn for his deaf one. He cannot feel proud of it, for it is to him a symbol of the utter failure of his cherished art."

"All diseases of the teeth," concludes Prof. B., "with the sole exception of the wasting of the alveolar processes, are curable in nearly every instance. We have learned to preserve the pulp, to cure the diseases of the teeth; to make roots that were troublesome, healthy and comfortable; we are making rapid progress in treating successfully atrophy of the alveolar processes; then am I claiming too much when I assert that the washing of hands by the 'best men,' is a good sign, and only precedes the hearty hand-shake over the appropriate and inevitable decline of mechanical dentistry."

---

*Davyum.*—A new metal christened by this title has recently been isolated by M. Serge Kern. It belongs to the platinum group.

*Diverse and Divers Views.*—As an example of the exceedingly diverse views expressed at Dental Associations on subjects in controversy, we make the following abstracts from the debate on a paper read before the American Dental Association, with the title, "Is the Dental Pulp Essential to the Integrity of the Tooth-Structure?"

"The presence of the tooth-pulp is necessary to preserve its (the tooth's) entirety."

"The pulp will reharden teeth softened by constitutional disease."

"The pulp has no connection with the dentine, and does not supply it with nourishment. The tooth is nourished by the pericementum."

"The dentinal fibrils can be drawn out of the dentinal canals."

"The pulp is a formative organ, and when that function is performed the tooth is complete, and needs little support."

"We over-estimate the importance of the pulp. Sometimes instead of being a benefit, it is a nuisance."

"It is the same thing to kill a pulp as to take off a leg, only on a smaller scale."

"I do not want any better practice than to cap freshly exposed pulps. I would rather cap than take the chances of extirpation."

"Oxychloride of Zinc is the most treacherous and deceitful thing one can use, an assassin in the dark. The risk of capping is ten times as great as devitalization."

"When a tooth aches violently, it (the pulp,) should be extirpated."

"When the pulp is destroyed, paralysis and atrophy ensue; in removing it the end is wounded, and some of the worst forms of facial neuralgia sometimes caused. It may even run back to the brain and set up a difficulty there."

"The dentine is supported by the pulp, and when it is removed the tooth will disintegrate."

---

*Eucalyptus as a Local Anæsthetic*—Dr. Horton, (Ohio State Dental Society,) speaks of the extract of eucalyptus as producing good results as a pain obtunder in sensitive dentine. A drop on a pledget of cotton is used. He thinks it the best of the local applications.

---

*Strychnia as a Substitute for Quinia.*—Mr. Geo. Brown, (*Chemist and Druggist,*) proposes the use of strychnine as a substitute for quinine, during the present high price of the latter. He thinks that in neuralgia it is quite equal to the great anti-periodic.

*Malaria.*—According to the U. S. census of 1870, malarial fevers are most fatal in 1st, Florida, Louisiana and Texas; 2nd, in Arkansas, Mississippi, Alabama, Georgia, Missouri, Kansas and Nevada: 3rd, in New Mexico, the Carolinas, Virginia, Tennessee, Kentucky, Illinois and Indiana; 4th, in New Eng-and, the Middle States, Wisconsin and Minnesota. Contrary to the common notion, there is considerable mortality from this cause in California. Localities subject to the inter-mixture of salt and fresh water are peculiarly prone to malaria. The use of impure drinking water seems to have some effect in promoting malarial disease; it is supposed by some that the presence of ozone and malaria are usually in inverse proportion, though this is denied. Malaria gains access to the system, mainly through the respiratory organs, is found most active about sundown and at night, will not pass over a large body of water, nor climb great heights, is dissipated by sunlight, and in part rendered innocuous by certain vegetable growths. According to some writers, suppurating wounds heal with difficulty in malarial subjects, and it is a formidable obstacle in the way of treating diseased or dead tooth pulps; and renders material modification of the treatment of pneumonias and other diseases necessary.

---

*Secondary Dentine.—Probabilities of After-destruction of Peripheral Cells of Pulp.*—Dr. M. S. Howe, in the N. Y. Odontological Society, March meeting, read a paper on *secondary dentine*, in which the ground was taken that "the expectation that a *peripheral plate of dentine* can and will be formed to close an orifice and *protect a pulp*, after the loss of a portion of the organ—i. e.—the pulp, is not well founded. He thinks that if any destruction of the cells upon the periphery of the pulp has occurred, "the conditions, whatever they may be, which induce the deposition of lime in the deeper portions of the pulp, must be in a greater degree morbid, and so uncertain as to give us little cause for cherishing great expectations. With the fact in view that the patient is greater than the tooth, will we not, if our premises are correct, be rendering the best service, in cases of exposure, with loss of a portion of the pulp, (the odontoblasts,) by removing the remainder of the pulp and thoroughly filling the cavity whence it come ?"

---

*Animal Magnetism.*— Dr. Kelly, (same society,) states that he spent three-quarters of an hour in digging out the root of a tooth, with the blade of a pen knife, his patient being magnetised and feeling no pain whatever.

